# Advances in Medicine: Photodynamic Therapy

**DOI:** 10.3390/ijms25158258

**Published:** 2024-07-29

**Authors:** David Aebisher, Jakub Szpara, Dorota Bartusik-Aebisher

**Affiliations:** 1 Department of Photomedicine and Physical Chemistry, Medical College of The Rzeszów University, 35-025 Rzeszów, Poland; 2 English Division Science Club, Medical College of The Rzeszów University, 35-025 Rzeszów, Poland; js126214@stud.ur.edu.pl; 3 Department of Biochemistry and General Chemistry, Medical College of The Rzeszów University, 35-025 Rzeszów, Poland; dbartusikaebisher@ur.edu.pl

**Keywords:** photodynamic therapy, photosensitizers, history of photodynamic therapy, in vitro, in vivo, nanotechnology, development, cancer therapies

## Abstract

Over the past decades, medicine has made enormous progress, revolutionized by modern technologies and innovative therapeutic approaches. One of the most exciting branches of these developments is photodynamic therapy (PDT). Using a combination of light of a specific wavelength and specially designed photosensitizing substances, PDT offers new perspectives in the fight against cancer, bacterial infections, and other diseases that are resistant to traditional treatment methods. In today’s world, where there is a growing problem of drug resistance, the search for alternative therapies is becoming more and more urgent. Imagine that we could destroy cancer cells or bacteria using light, without the need to use strong chemicals or antibiotics. This is what PDT promises. By activating photosensitizers using appropriately adjusted light, this therapy can induce the death of cancer or bacterial cells while minimizing damage to surrounding healthy tissues. In this work, we will explore this fascinating method, discovering its mechanisms of action, clinical applications, and development prospects. We will also analyze the latest research and patient testimonies to understand the potential of PDT for the future of medicine.

## 1. Introduction

### 1.1. Advances in PDT Research in Ancient Civilizations

Photodynamic therapy (PDT) and its history may go back further than you might think. The first mentions and evidence of the use of this therapy date back several thousand years. In ancient civilizations such as Egypt, India, or China, where, by appropriately combining the rays of sunlight with plants and vegetables, it was possible to obtain appropriate reactions. Thanks to this special combination, they treated diseases such as:

- Psoriasis, which manifests itself as red spots and silvery scales resulting from the excessive multiplication of skin cells;
- Vitiligo, i.e., a disorder of melanin production, resulting in white spots all over the body;
- Rickets, caused by vitamin D deficiency [1,2,3].

The method used by the ancient Egyptians to pigment de-pigmented skin lesions was to produce a special powder, which included, among others, parsnip, parsley, or St. John’s wort, and then it was applied to designated areas on the skin and exposed to light, which caused skin pigmentation [4].

In China, these methods were completely different. An innovative therapy was introduced to these areas by Lingyan Tzu-Ming in the first century BC. However, after four centuries, phototherapy became a more ritual act, where people, after soaking green paper with red dye and then exposing it to light, consumed it, believing in its healing properties [5,6].

Historical references to the described therapy can also be found in ancient Greece, where various types of diseases were treated through the full exposure of the human body to sunlight—in other words, heliotherapy. Stopping in ancient Greece, you can come across the famous philosopher Herodotus, who is considered the father of heliotherapy and was the first to preach about the enormous importance of exposing the human body to sunlight and the health benefits of this activity [1].

### 1.2. Advances in PDT Research in the 18th and 19th Centuries

Further references to this therapy can be found in Europe and, more specifically, in France, where at the turn of the 18th and 19th centuries, light treatment had a positive effect in the treatment of diseases such as scurvy, tuberculosis, rheumatism, and muscle weakness [4].

For the 19th century, it is worth mentioning the person of Arnold Rikie, a Swiss physicist and a profound defender of natural medicine, who tried to restore the healing effects of light. His efforts contributed to the establishment of the National Medical Institute in Slovenia in 1855. His famous saying was: “Water is good, air is better, but light is the best”, which he tried to prove over 50 years of his work [5].

However, the greatest development of photodynamic therapy took place in the 20th century, when the Danish physicist, Niels Finsen, who was born in the Faroe Islands and from a young age was fascinated by the effects of light and its contrasts in the Arctic Circle, decided to devote his life to studying the effects of sunlight on living organisms. This led him to initiate phototherapy for pityriasis vulgaris, but this was not his greatest achievement.

In 1893, he published a groundbreaking work that described the successful treatment of chickenpox using red light, which prevented the vesicles from suppurating. In 1903, he received the Nobel Prize for his achievements in the field of photodynamics. Interestingly, Finsen himself, already in 1896, headed the world’s first Institute of Phototherapy, which was founded in Copenhagen [2,4].

A great advocate of this therapy was Princess Alexandra, who popularized this discovery throughout England. She encouraged doctors at the Royal Hospital, with which she was closely associated, to use phototherapy more often in the treatment of various diseases [5].

The concept of a dead cell that is induced by light was first documented by Oskar Rabb, who was a medical student working under the supervision of Professor Hermann von Tappeiner in Munich. The discovery itself took place during Rabb’s research on the effect of acridine on malaria and the connection between protozoa. It turned out that the combination of red acridine and light produces a lethal combination that causes the death of Infusoria (a species of protozoan). It is worth adding that the result of this experiment was accidental because the results were obtained during a storm and the scientist himself then carried out work to strengthen the obtained effect [1].

Rabb himself discovered (probably unknowingly) the fluorescence effect. He concluded that it was not the light in this experiment that caused the toxicity but the fluorescence product. Shortly later, he formulated a hypothesis, namely, that the effect obtained was the result of the transfer of light energy to a chemical substance, in a similar way to how photosynthesis is carried out in plants [6].

### 1.3. Advances in PDT Research in the 20th Century

After some time, Herman Von Tappeiner began research with a dermatologist named Jesionek. Their joint work resulted in the results of clinical trials that proved the action of eosin as a photosensitizer in the treatment of skin cancer, dandruff, and genital warts. Later, more precisely in 1904, it was proven that oxygen was necessary for this process [5].

One of the breakthrough moments in the history of photodynamic therapy is the discovery and development of hematoporphyrin. It was produced by Scherer in 1841 after treating a blood sample and treating it with sulfuric acid. Then, in 1867, the fluorescence and spectrum of this compound were described, and the scientist who accomplished it was Thudi-chun.

The photodynamic properties of this compound were examined in the years 1908–1913, and the research was carried out on various objects, including erythrocytes, mice, guinea pigs, and even humans. The first experiment on the toxicity of hematoporin to the human body took place in 1912, when the German doctor Friedrich Meyer Betz administered 200 mg of the test substance intravenously, after which many changes were observed. Due to the phototoxic compound, the patient suffered from excessive swelling and significant skin lesions after exposure to the sun for approximately 2 months after the experiment [6].

Photodynamic therapy is widely used in the treatment of cancer. A milestone in this aspect was achieved in 1924, when a French researcher, Policard from Lyon, observed a characteristic red color resulting from the presence of hematoporphyrin, which appeared after exposure to Wood’s lamps emitting UV rays. The characteristic color of sarcoma cells giving a characteristic luminescence was then discovered [1,4].

The next discoveries in the field of photodynamics date back to 1942, when two scientists from Germany, or more precisely from Berlin, named Auler and Banzer, decided to investigate and evaluate the process of accumulation and fluorescence of porphyrins administered exogenously to the body [4]. The results of this experiment were surprising; namely, it was proven that hematoporphyrin accumulates both in primary tumors and metastases but also in lymph nodes, and their exposure to light gave positive results [6].

In 1948, Figge showed that hematoporphyrin accumulates selectively, and it was then recognized as a potential tool for cancer diagnosis. Rasmussen-Tiurdal, Ward, and Figge in 1955 studied this substance for its accumulation and localization in various types of tumors. However, a permanent accumulation of doses of hematoporphyrin proved impossible, given its high risk of phototoxicity [1].

Although research on photodynamic therapy has been dynamically conducted, the history of photodynamic therapy, or more precisely, its first application, took place in 1966, when Lipsonn and his team announced the first use of HpD (a derivative of hematoporphyrins) and local exposure with xenon arc lamps. The tumor treated with this therapy was a large, recurrent, ulcerated breast tumor, which returned a few weeks after a series of radiation treatments, but it gave scientists evidence of the body’s response to the innovative therapy.

However, in 1966–1967, other reagents were tested as sensitizing agents, including methylene blue, with some success. Namely, there was significant tumor disintegration and healing of wounds resulting from the presence of the unwanted lesion [1].

The next step that revolutionized the photodynamic therapy community was the feat that was achieved in 1975, when Dougherty and his colleagues, who conducted their research at the Roswell Park Institute in Buffalo, managed to completely cure a tumor in an animal after the administration of HpD and subsequent activation with red light. Studies have shown that in as many as 48% of the tested mice the mammary tumor will completely disappear [1]. This stimulated scientists from all over the world to intensify their attempts to transfer their success to the human body. 

Kelly from Saint Mary’s Hospital in London showed that human bladder tumor cells, after transplantation into mice, could be destroyed using photodynamic therapy.

A year later, he decided to transfer his success to a larger scale when he began treating a patient with recurrent, extensive bladder cancer after many unsuccessful treatment attempts, including radiotherapy or chemotherapy. A mercury lamp to activate HpD and a quartz rod were used to attempt to destroy the tumor. The results of the experimental therapy turned out to be surprisingly good: in the irradiated area, scientists could observe complete tumor necrosis and, equally importantly, there were no signs of the side effects of irradiation on the patient’s entire body. It was a huge success and a great day in the history of PDT [1].

In 1978, however, Dougherty examined 25 patients with a total of 113 skin lesions of a cancerous nature, and all of them were subjected to photodynamic therapy. The effects were surprisingly good: only two areas of the human body remained unaffected by the treatment and, out of 113 lesions, as many as 98 skin lesions were completely destroyed. Although some side effects could be noticed, they could be eliminated by using an appropriate time interval between exposures.

Shortly thereafter, doctors noticed that PDT could be used in bronchoscopy or, more specifically, in the treatment of lung tumors that were inoperable and resistant to various other forms of therapy, such as radiotherapy. The first description of these activities took place in 1982 by a scientist named Hayata; the reactions he described were significant in most patients, but only one in 14 patients achieved complete recovery [1,3].

Oncologists treating esophageal cancer have noticed a huge opportunity for photodynamic therapy. In 1984, McCaughan and his team of specialists treated seven patients with esophageal tumors that made swallowing very difficult. A positive effect of the therapy was achieved in two patients who were able to swallow freely for the next 11 months [1].

A year later, Hayata began treating superficial tumors of the esophagus and early stages of stomach cancer. Both of these attempts ended with considerable success, as a large number of patients undergoing therapy became free of ailments after treatment [1].

It is estimated that before 1996 several tens of thousands of people with cancer were treated around the world using photodynamic therapy and, what is worth adding, with very satisfactory effectiveness.

### 1.4. Advances in PDT Research in the 21st Century 

As for Poland, compared to the rest of the world, the first work on PDT began in 1984 at the Central Clinical Hospital of the Military Medical Academy in Warsaw and was carried out on animals [4]. In 2005, with the establishment of the Polish Society of Laser Medicine and Photodynamic Therapy, active multicenter cooperation in the field of photodynamic methods became a fact. The society has about 100 members and has organized two national congresses: in 2006, 100 people participated and, in 2008, about 400.

One of the oldest Photodynamic Diagnostics and Therapy Centers is located in Bytom. This facility has been conducting clinical research for over 10 years in fields such as dermatology, gastroenterology, laryngology, pulmonology, gynecology, and orthopedics. This center was founded in 1998 as a result of the cooperation between various specialists involved in intensive clinical activities. It uses the Onco-LIFE autofluorescence imaging system (light-induced fluorescence endoscopy; Xillix, Richmond, BC, Canada), which shows optical differences between healthy and neoplastic tissue, correlating them with the histopathological degree of cancer lesions, which is an innovative diagnostic approach. In addition, it uses fluorescence spectroscopy to analyze spectral differences in real time while monitoring photodynamic treatment, which allows increasing the sensitivity and specificity of a medical diagnosis in clinical practice.

Over the last 25 years, doctors at the center have performed 13,287 procedures using PDT (based on available data from 2019), including autofluorescence gastroscopy, colonoscopy, bronchoscopy, cystoscopy, and dermatological examinations. During this time, PDT therapy was also performed on 5203 patients with various precancerous and cancerous lesions of the digestive tract, respiratory tract, urinary tract, and skin [7,8].

The rest of this work focuses on the development of photodynamic therapy in the years 1990–2023 and the broad branches of PDT in the treatment of various types of diseases. The mechanism of action of the therapy from a biochemical perspective, together with photosensitizers, and the prospects for broader research in the field of PDT, are also discussed.

## 2. Materials and Methods

A review of sophisticated articles was necessary to properly prepare the work. An analysis of scientific works was carried out on the PubMed and Google Scholar databases. 

To properly conduct the review, clinical studies, review articles, and preclinical studies were taken into account that covered photodynamic therapy with particular emphasis on its history, mode of action, areas in which it is most widespread, and the components necessary for its operation.

The inclusion criteria included papers that placed particular emphasis on photodynamic therapy. The exclusion criteria, however, were works that were in no way related to photodynamic therapy or their substantive content differed from the desired quality standards.

The first step in the research analysis process was the selection of appropriate studies, taking into account the inclusion and exclusion criteria; articles that passed the first stage were subjected to a detailed analysis in terms of their substantive content. During the selection, great emphasis was placed on the history of photodynamic therapy, in particular time frames, the use of photodynamic therapy in the clinic, photosensitizers with their origin and application, and prospects for further research and development of this therapy.

When analyzing the works, it was necessary to specify several reference points that the work would focus on. They included the broadly understood history of photodynamic therapy along with its development and prospects for further years, in vivo and in vitro applications, photosensitizers in a broad sense, and the entire mechanism effects of photodynamic therapy, with particular discussion of each of its necessary components.

After prior analysis, the information received was sorted in order from ancient times to the present day. Specific sections were sorted in the appropriate order to harmoniously discuss and possibly exhaust the topic of photodynamic therapy. By strictly applying the above criteria, it was possible to shed new light on photodynamic therapy through its in-depth analysis.

### 2.1. Mechanism of Action of PDT

From a molecular point of view, the effectiveness of photodynamic therapy (PDT) depends on the interaction of three key factors:

- Photosensitizer—a chemical substance that, when exposed to the light of an appropriate wavelength, becomes active and generates reactive oxygen species (ROS), which are responsible for killing cells. Photosensitizers may be organic or inorganic chemical compounds. Organic photosensitizers, such as chlorophyllins and porphyrins, are most often used due to their ability to absorb light in the visible and near-infrared range.
- Light of the appropriate wavelength—to activate the photosensitizer, it is necessary to use the light of a specific wavelength that matches the absorption range of the photosensitizer. The most commonly used light source is a laser or LED-emitting light of a specific wavelength, the absorption of which by a photosensitizer leads to the generation of ROS.
- Dissolved oxygen in cells—the presence of oxygen in cells is crucial for the formation of reactive oxygen species during PDT. The PDT process involves the activation of a photosensitizer by light, which leads to the conversion of oxygen in the cell into a singlet oxide, which then attacks and destroys cancer cells or pathogens. The lack of oxygen in cells (hypoxia) may limit the effectiveness of PDT; therefore, the availability of oxygen is an important factor influencing the effectiveness of the therapy.

The interaction of these three factors is crucial to achieve the desired effect of photodynamic therapy, i.e., the effective destruction of, among others, cancer cells or pathogenic microorganisms [7].

The photodynamic process can occur through two different mechanisms, but both depend on the presence of oxygen in the cells. The first stage of both mechanisms is similar: after entering the cell, the photosensitizer is irradiated with the light of the appropriate wavelength, which causes it to jump from the S_0_ energy state to the S_1_ excited state by absorbing light photons. Then, part of the energy is emitted in the form of a fluorescence quantum, and the remaining part directs the photosensitizer molecule to the triple T_1_ state, which is the proper form of the compound with therapeutic properties.

In the triple T_1_ state, the photosensitizer can convert ordinary oxygen molecules into a reactive form of oxygen. This reactive oxygen species can react with nearby biological molecules such as lipids, proteins, or nucleic acid, leading to structural and functional damage to cells. The resulting damage is responsible for the destruction of cancer cells or pathogenic microorganisms, which leads to the therapeutic effect of photodynamic therapy. In the absence of oxygen in the cell, the photosensitizer may react with other substrates, leading to the formation of other reactive oxygen species, which may also have a therapeutic effect. However, the reaction with oxygen is the main mechanism of action of photodynamic therapy [8,9].

The first reaction mechanism in photodynamic therapy is the transfer of hydrogen or electron between the T_1_ triple state of the photosensitizer and the pathogenic tissue. For example, let assume that the type of tissue will be cancerous tissue. As a result of this process, free radicals are formed, both with the anionic radicals of the photosensitizer and with the substrate. The oxygen molecules are also exposed to electrons, maintaining their basic electrical state. This process leads to the formation of reactive oxygen species (ROS), which in turn initiates subsequent reactions, creating an ROS cascade.

The cascade of these reactions leads to a phenomenon called oxidative stress. This is a situation in which excess ROS in the body exceeds the ability of cells to neutralize them. As a result, cancer cells are damaged at the structural and functional levels, which may lead to their death. Oxidative stress also affects other cellular elements, such as proteins, lipids, and nucleic acid, which may affect various physiological processes.

As a result, photodynamic therapy stimulates the process of apoptosis or necrosis in cancer cells, which leads to the inhibition or reduction in tumor growth. The therapeutic effect of this method is based on the use of the ability of the photosensitizer to react with oxygen to generate ROS, which is crucial for the destruction of cancer cells [10,11].

Another possibility is to transfer energy directly to the oxygen molecule through the photosensitizer. This process, known as direct transfer, occurs when photosensitizers have the same electron spins. These spins are a property of molecules that determine their quantum state. When the spins are aligned, it is possible to directly transfer energy from the photosensitizer to the oxygen molecule.

**Figure 1:**
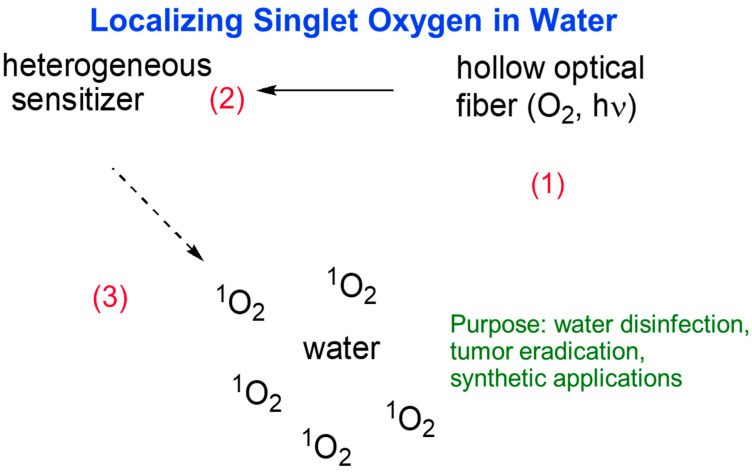
Generation of ^1^O_2_ in water.

As a result of this energy transfer, singlet oxygen (^1^O_2_) is generated, which is characterized by exceptionally high oxidizing properties (Figure 1). Singlet oxygen is extremely reactive and can lead to cell damage at the structural and functional level. Its ability to oxidize organic substances makes it an effective anticancer agent in photodynamic therapy [10,12].

During the photodynamic reaction, various types of reactive oxygen species can be generated, such as hydrogen peroxide (H_2_O_2_), hydroperoxyl (HO_2_^•^), nitric oxide (NO^•^), as well as hydroxyl radicals (^•^OH). These molecules are very chemically reactive and can cause photodamage at various levels in the cancer cell.

In response to these damages, the cell can activate various defense mechanisms. One such mechanisms is apoptosis, i.e., the process of programmed cell death. In apoptosis, the cell is removed in a controlled manner, leading to the fragmentation of the cell into smaller parts, called apoptotic bodies. This process occurs in response to a variety of internal or external signals that indicate the need for cell elimination. Apoptosis is beneficial to the body because it allows the removal of damaged or unwanted cells without causing inflammation, which helps maintain tissue homeostasis.

In turn, cell necrosis, also known as necrosis, is a process of uncontrolled cell death. It may occur as a result of extreme environmental conditions, such as mechanical trauma, toxic chemicals, or high temperature. In necrosis, the cell undergoes rapid disintegration, which leads to the release of its contents into the surrounding environment. This process can cause inflammation and damage to the surrounding tissues, which can be harmful to the body [13].

### 2.2. Photosensitizers

Photosensitizers are key ingredients in photodynamic therapy (PDT). These are chemicals or dyes that have the ability to absorb the light of a specific wavelength. For a photosensitizer to be effective in PDT therapy, it must meet several important features:

- First, thermostability is key because the photosensitizer must be stable during storage and application to maintain its therapeutic properties.
- Improper storage or loss of stability may affect the effectiveness of the therapy.
- Secondly, the photosensitizer should not be susceptible to interactions with other chemicals present in the body that may reduce its effectiveness or affect the PDT process in an undesirable way.
- The wavelength of light that the photosensitizer is able to absorb is also crucial. The optimal wavelength depends on the type of tissue and type of pathology but usually does not exceed 800 nm. When this value is exceeded, light penetration may be limited, which may reduce the effectiveness of the therapy.
- Furthermore, the photosensitizer should be highly soluble in the body’s tissues so that it can be easily distributed and accumulated in the treatment area. This enables the effective penetration of the photosensitizer into the target tissue.
- Low price and simplicity in synthesis are also important because they enable the mass production and widespread use of PDT therapy.
- Ultimately, high selectivity for tumor tissues is a key feature that allows the precise destruction of tumor cells while minimizing damage to healthy tissues. It is this selectivity that allows effective therapy with minimal side effects [14,15,16,17].

In the early 1970s, Dr. Thomas Dougherty made a breakthrough with the discovery of photosensitizers based on hematoporphyrin, also known as HpD. Hematoporphyrin was purified and chemically modified, giving rise to the first photosensitizer that was used in photodynamic therapy.

HpD was the first photosensitizer to enter public use, opening the way for further research and development in this field. However, first-generation photosensitizers such as HpD had their limitations and drawbacks. One of the main problems was their low chemical purity, which made their clinical use difficult. Additionally, some of these compounds had limited effectiveness in killing cancer cells and may also cause undesirable side effects.

Therefore, there was a need to find new, better photosensitizers that could be more effective and safe in clinical applications. The development of new photosensitizers, especially those with higher chemical purity and greater selectivity for cancer cells, has become the goal of a great deal of research and numerous experiments in the field of photodynamic cancer therapy [18,19,20,21].

In the 1980s, work began on second-generation photosensitizers, which were intended to improve some of the limitations of first-generation photosensitizers. One of the key features of the second-generation photosensitizers was an increase in the efficiency of singlet oxide production, which made them more effective in destroying cancer cells. In addition, the second-generation photosensitizers also had deeper tissue penetration, which allowed the light to reach areas deeper in the body. An important advantage of these compounds was also their higher chemical purity compared to first-generation photosensitizers, which made them more stable and less susceptible to degradation.

However, second-generation photosensitizers also had their drawbacks. One of the main disadvantages was their limited solubility in water, which made their intravenous administration difficult and required other application methods. Despite these limitations, researchers continued to search for new substances that could be more effective and more versatile in clinical applications.

During this time, many potential substances were discovered, but one of the key discoveries was 5-aminolevulinic acid (ALA). It turned out that ALA is a precursor of protoporphyrin IX, which is a key ingredient in the production of photosensitizers. This discovery was of great importance in the field of photodynamic therapy because it enabled the effective introduction into the body of substances that could be used for therapeutic purposes [22,23,24].

Nowadays, the development of third-generation photosensitizers brings new approaches to photodynamic therapy based mainly on the synthesis of substances with a higher degree of affinity towards cancer tissues. One of the main goals of this research was to increase the selectivity of photosynthesizers, so that they can reach cancer cells more precisely while minimizing the effect on healthy tissues.

In recent years, various modifications have been used to increase the effectiveness of photodynamic therapy. One example of such modifications is the synthesis of photosynthesizers with LDL lipoproteins. This is particularly important due to the fact that cancer cells need a large amount of cholesterol to synthesize their cell wall, which means that they have a large number of LDL receptors on their surface. Using this property, photosynthesizers can be effectively delivered to cancer cells by combining them with LDL lipoproteins. Thanks to this, photosynthesizers can be more concentrated in cancerous areas, which increases the effectiveness of photodynamic therapy and minimizes the effect on healthy tissues. This is just one example of innovative approaches that aim to improve the effectiveness and precision of photodynamic therapy [25,26].

Below (Table 1) is a collection of the most popular photosensitizers, including their clinical use and manufacturer [27,28,29,30,31,32,33,34,35,36].

## 3. The Development of PDT Clinical Applications

**Figure 2:**
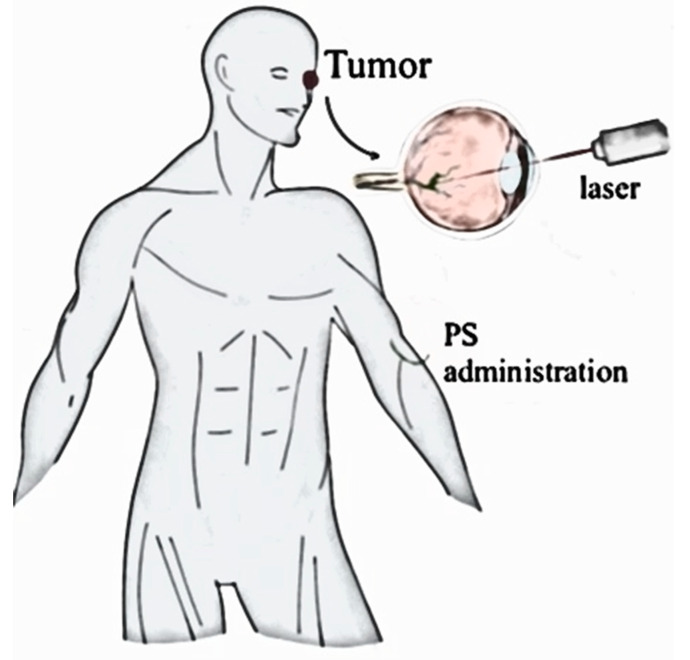
An example of PDT application. The graphic shows intravenous administration of a photosensitizer and localized application of light where the tumor is located, including the eyes.

From the 1990s to 2023, photodynamic therapy (PDT) has undergone significant development, encompassing many key aspects that have influenced its use and effectiveness in medicine. During this period, researchers focused on the synthesis and testing of new photosensitizers, trying to discover compounds that would be more selective for cancer cells, less toxic to healthy tissues, and better penetrate deeper tissue layers. New light sources were introduced, such as lasers and light-emitting diodes, which allowed for more accurate and deeper light delivery, as well as optical fibers, which opened the way to difficult-to-reach areas of the body (Figure 2).

The development of photodynamic diagnostics (PDD) was a parallel path, enabling the more precise detection and treatment planning of cancer by using the fluorescence of activated photosensitizers. Photodynamic therapy has also gained application outside oncology, including dermatology, ophthalmology, and gynecology, as well as in the treatment of inflammatory skin diseases and vascular disorders. Innovations in PDT have also included integration with other forms of therapy, such as chemotherapy, radiotherapy, and targeted therapies, which have increased treatment effectiveness while minimizing side effects.

Interest in personalizing photodynamic therapy has increased, prompting specialists to tailor treatment approaches to individual patient needs, taking into account factors such as the type and location of the tumor and the patient’s genetics and previous treatment. All these activities contributed to progress photodynamic therapy, opening new perspectives in the treatment of many diseases [37,38].

In 1991, it was discovered that photodynamic therapy (PDT) induces apoptosis, or programmed cell death, which explains its effectiveness in a wide range of applications. The main cause of apoptosis is believed to be mitochondrial damage. Initial photooxidative damage initiates a series of reactions, including direct cytotoxicity in the tumor microenvironment. These processes, combined with endothelial cell damage, lead to microvascular collapse and cell death caused by hypoxia [39].

The first official approval for the use of photodynamic therapy (PDT) was issued in Canada in 1993 for Photofrin, intended for the treatment of bladder cancer. Over time, Photofrin also became licensed in other countries for the treatment of various types of cancer, including lung, bladder, cervix, and esophagus [1].

In 1995, Lightdale and his team published the results of a study in which they compared the effectiveness of photodynamic therapy (PDT) using Photofrin and thermal Nd:YAG ablation in the treatment of partially blocked esophageal tumors. This study was prospective, randomized, and multicenter. The results showed that both methods provided similar relief from dysphagia symptoms, but the effects of PDT were more durable over time (32% of patients had improvement after one month compared to 20% with thermal Nd:YAG ablation). Furthermore, more patients achieved a complete response to PDT therapy than to thermal Nd:YAG ablation. PDT also required fewer procedures than thermal ablation (average 1.5 vs. 2.4) and had a lower risk of esophageal perforation (1% vs. 7%). These observations led the FDA to approve the use of Photofrin in the US for the treatment of advanced esophageal cancer. This important event opened the way to the approval of PDT in other countries and its use in various indications [40].

In a study conducted by Mladen Korbelik and colleagues in 1996, the long-term effects of PDT on tumor growth in healthy Balb/C and immunodeficient mice were examined. Although the short-term tumor response to PDT with Photofrin appeared similar, the long-term effects varied significantly. They found that tumor recurrence was more common in immunocompromised mice. However, this effect was reversed after bone marrow transplantation from immunocompetent Balb/C donors. These results suggest that, although the direct action of PDT can destroy most of the tumor, an immune response is necessary to eliminate surviving cells. This study proved that, despite the wonderful tool that is photodynamic therapy, a well-functioning immune system is necessary for full health [41].

In 1998, Photofrin was approved for the treatment of early-stage nonseminomatous squamous cell carcinoma of the lung. There are currently 50 photodynamic therapy (PDT) studies with Photofrin listed on the clinical trials website. These studies cover a variety of cancer types, such as inoperable papillary biliary carcinoma, recurrent high-grade glioma, advanced pancreatic cancer, head and neck cancer, epithelioid peritoneal mesothelioma, and advanced rectal cancer [42].

In 1999, Korbelik and his team discovered that PDT causes the production of immune cells that respond to the presence of cancer. These cells could be found in lymphatic areas distant from the treated tumor area at various times after PDT. This provided a scope for further research into the synergy of photodynamic therapy with combined immunotherapies [43,44].

In 2000, Verteporfin, also called Visudyne, was approved by the FDA for the treatment of macular neovascularization caused by wet age-related macular degeneration (AMD). It is supplied as a frozen liposomal preparation that can be rehydrated and administered intravenously. Despite the misleading name of “monoacide derivative of the A-ring of benzoporphyrin”, its chemical structure is chlorine, and the main absorption peak occurs at 690 nm [45].

Foscan, also known as temoporfin or m-tetrahydroxyphenylchlorin (mTHPC), was approved by the European Medicines Agency (EMA) in 2001 for the treatment of advanced squamous cell carcinoma of the head and neck. Although attempts were made to introduce it to the US market, the FDA rejected the application for approval in 2000. Clinical studies on Foscan include its use in the treatment of inoperable bile duct cancer, inoperable nonseminoma squamous cell carcinoma of the lung, and nasopharyngeal cancer. It is administered intravenously at a dose of approximately 0.15 mg/kg, usually 96 h before light exposure. Since Foscan does not dissolve in water, it is administered dissolved in ethanol and propylene glycol. It is activated by light with a wavelength of 652 nm, and its advantages are high efficiency and light absorption at this wavelength, characteristic of chlorin. However, due to its powerful strength, Foscan may cause damage to the tissues surrounding the tumor and has a risk of skin burns at the site of drug administration. Skin hypersensitivity after treatment lasts for about 2 weeks [42,46].

In 2002, PDT protocols were revised to optimize the targeting of both vascular regions and tumor cells. It was discovered that administering photosensitizers at various intervals before activation with light (fractionated dose of the PDT drug) is the most effective way of affecting both tumor blood vessels and tumor cells. PDT protocols based on fractionated drug doses have been reported to provide better therapeutic effects than single-use regimens such as antivascular or antitumor PDT and may lead to the long-term control of tumor growth. This is further evidence that targeting several compartments is the most effective way to combat cancer [47].

In 2004, Talaporfin sodium (a derivative of chlorin(e6)) received approval in Japan for use in photodynamic therapy for early-stage cancer. Its chemical structure, also known as NPe6, is the tetrasodium salt of chlorin e6 mono-L-aspartate. In 2007, Smith and a team of scientists conducted research that showed that the correct structure included aspartic acid attached to the COOH position of the side chain at the 15^2^ position. It is a water-soluble chlorine, administered intravenously at a dose of 1 mg/kg, and is quickly removed from the body, which minimizes problems with skin photosensitization. Clinically, the interval between drug administration and light exposure ranges from 0.25 to 4 h. Clinical trials include, among others, inoperable liver cell cancer and colorectal cancer metastases to the liver [48,49].

Photolon (or Fotolon) is a mixture of chlorin(e6) and polyvinylpyrrolidone, formulated in a 1:1 ratio. It was approved in Belarus in 2001 and in Russia in 2004. Photolon has been subjected to clinical trials in various types of cancer, mainly in Russia, including skin cancer, cervical epithelial dysplasia, lung cancer, disseminated forms of melanoma, and brain tumors (both primary and metastatic, during surgery). Typically, Photolon is administered intravenously at a dose of 2–2.5 mg/kg, and after 3 h, it is irradiated with a laser with a wavelength of 662 nm, depending on the location of the tumor, at doses from 50 to 600 J/cm^2^ [50,51].

Photosens is a water-soluble mixture of an aluminum compound with sulfated phthalocyanines, which differ in the degree of sulfation. It received a number of regulatory approvals in Russia between 2001 and 2008. It is administered intravenously to patients at a dose of 0.3 to 0.8 mg/kg and then subjected to several laser irradiations with a wavelength of 675 nm, performed between 24 and 72 h after drug administration. Each exposure has a dose of 80–100 J/cm^2^, which gives a total of up to 600 J/cm^2^. The clinical trials conducted included various types of cancer, such as basal cell carcinoma, lung cancer, head and neck cancer, stomach cancer, esophageal cancer, pleural sarcoma, and breast cancer metastases [52,53].

ALA BF-200 gel, known as ALA 10%, was approved by the European Medicines Agency in December 2011 for the treatment of mild to moderate actinic keratosis on the face and scalp. Later, in the following years, the scope of use of this preparation was extended to include other types of skin cancer. In 2016, it was approved for the treatment of lesions in a broader cancer field. In 2017, it covered basal cell carcinomas, and, in 2020, actinic keratosis on the limbs and trunk/neck. ALA Gel 10% was also approved by the US Food and Drug Administration in 2016 for use in conjunction with a red light lamp for the treatment of actinic keratoses on the face and scalp [54].

In China, Hemoporfin, a substance with the chemical structure of hematoporphyrin mono-methyl ether, was developed and approved for use in PDT of port wine-type skin pigmented lesions. In 2012, it received regulatory approval in China. Clinical trials are currently being conducted in this field, especially in children. Moreover, the possibility of testing the effectiveness of Hemoporfin in the endoluminal treatment of cancer is being considered [42].

Tookad is a name that refers to two different compounds developed by scientists from the Weizmann Institute in Israel—Avigdor Scherz and Yoram Salomon. The original Tookad, designated WST-09, was a bacteriopheophorboid derivative containing palladium but was insoluble in water and was administered intravenously dissolved in Cremophor EL. In 2005, scientists developed a new version of Tookad called WST-11 or Stakel and later Pa-deliporfin, which was water-soluble. Both Tookad compounds turned out to be fast-acting substances that were activated mainly in blood vessels, which allowed their use in treatments targeting blood vessels (VTP). Clinical studies on Tookad included, among others, the treatment of recurrent prostate cancer by intravenous administration followed by light irradiation. WST-11 received approval from the European Medicines Agency in 2017, is currently on the market in Mexico, and is under consideration by the FDA. Clinical trials confirmed the effectiveness and safety of Tookad VTP, which was proven in multicenter clinical trials on 413 patients from 10 countries. Tookad is also currently being studied in the treatment of other types of cancer, such as renal cell carcinoma, as well as in patients at low and intermediate risk of prostate cancer [42,55].

The year 2018 brought surprising discoveries in the field of combining PDT with immunotherapy when Im and co-workers developed nanoparticles containing an admixture of Ce6, azobenzene-glycol, chitosan (GC), PEG, and mesoporous silica, responsive to hypoxia (CAGE), intended to enhance cancer immunotherapy (CIT) with PDT by modifying dendritic cells and destroying cancer cells. The efficient generation of ROS by CAGE during laser irradiation was confirmed by SOSG. Biocompatibility and photosensitizing toxicity tests of carriers were carried out in vitro on the murine melanoma cell line (B16-F1) using light with a wavelength of 660 nm and power of 150 mW/cm^2^ for 15 min. In vivo studies on B16-F1 tumor-bearing mice showed the effective inhibition of tumor growth in mice treated with CAGE and irradiated at 660 nm (power 200 mW/cm^2^ for 15 min) [56].

In 2019, Mei and other researchers conducted a study in which they used 5-ALA (5-aminolevulinic acid) with or without hyperbaric oxygen therapy (HBO) to treat human A431 cell squamous cell carcinoma in vitro. The cells were irradiated for 8 days after 5-ALA and HBO therapy. The results showed that both methods caused a decrease in cell proliferation depending on the concentration of 5-ALA. In particular, a synergistic effect of the combination of 5-ALA and HBO was observed, especially at higher 5-ALA concentrations. An analysis of apoptosis-related proteins showed increased Bax and caspase 3 protein levels and decreased Bcl-2 protein levels in A431 cells, suggesting the activation of the mitochondrial apoptosis pathway. Additionally, a dual treatment with 5-ALA and HBO reduced the production of reactive oxygen species (ROS) and promoted autophagy [57]. In the same year, 2019, research was carried out to optimize photodynamic therapy and, more precisely, to provide one of its essential components—oxygen. The problem seems to be solved by intelligent nanoparticles (NPs) developed by Cao and colleagues, aimed at overcoming the limitations associated with the dual treatment with PDT and PTT. These nanoparticles consisted of an MnO_2_ nanoplate that was decorated with Cu_2_-xS nanocrystals (MnO_2_/Cu_2_-xS) on the surface and were then loaded with siRNAs (small interference RNAs), ultimately forming MnO_2_/Cu_2_-xS-siRNA. In an acidic environment, MnO_2_ was reduced to the Mn^2+^ ion, which caused the decomposition of H_2_O_2_ to O_2_, which facilitated the fight against hypoxia in the tumor. Reduced Mn^2+^ ions significantly increased MRI contrast, and Cu_2_-xS acted as a strong PA (photoacoustic) and PT (thermal) imaging agent, leading to accurate trimodal imaging and tumor detection [58,59].

The possibilities of using PDT as an antiviral therapy are also being investigated, including in the context of reducing the titer of the SARS-CoV-2 virus, which is the factor causing COVID-19 disease. The first scientific research on this topic was published in 2022, but the methods still require further research [60].

### 3.1. The Development of Antibacterial PDT

Antibacterial photodynamic therapy is one of the newest branches of photodynamic therapy. Its main goal is to treat local infection foci, with particular emphasis on post-operative wounds and burns.

Its main task is to destroy cells through photodynamic inactivation, which is to prevent them from developing resistance. Bacteria that showed the expected response to therapy, min. *Pseudomonas aeruginosa* (*P. aeruginosa*), *Staphylococcus aureus* (*S. aureus*), *Acinetobacter baumannii*, and *Klebsiella pneumoniae*, were often difficult to treat in the long term; however, through the use of APDT, the desired effects were achieved in the long term [61].

Photodynamic therapy is a promising method of treating infected skin and mucosal wounds, trophic ulcers, and diabetic ulcers. The activation of the photosensitizer by light leads to the repeated oxidative destruction of various subcellular structures of pathogens. Unlike antibiotics, photodynamic therapy does not lead to the development of bacterial resistance and does not affect the patient’s normal microflora. The effectiveness of the therapy depends on the properties of the photosensitizer molecules, such as molecular weight, charge, and photodynamic properties. In the case of Gram-negative bacteria, the effectiveness of photodynamic inactivation depends on the use of low-molecular-weight polycationic photosensitizers. Bacterial biofilms constitute an additional obstacle to photodynamic therapy, but appropriate photosensitizers can contribute to the effective inactivation of pathogens even in the presence of biofilm [62].

In 2019, an extremely simple and effective protocol was developed for the simultaneous use of Gram-positive bacterial differentiation and photodynamic antibiosis, using water-soluble near-infrared emission AIEgen (TTVP). With excellent uniformity in culture media, strong electrostatic interaction with Gram-positive bacteria, and AIE features, TTVP can quickly distinguish Gram-positive from Gram-negative bacteria using fluorescence technique. Just a few seconds of quick incubation is enough, and the staining procedure without the need for rinsing becomes possible.

TTVP can also effectively generate ROS, which is significantly superior to Rose Bengal and Ce6, which are two of the most effective and popularly used PSs. Together with its strong absorption in the visible light region and rapid ability to insert into the membrane, TTVP at concentrations as low as 0.125 µM is capable of completely inactivating Gram-positive bacteria when irradiated with white light. It is an extremely effective, optically indirect antimicrobial agent.

More importantly, TTVP shows significant efficacy in in vivo antibacterial photodynamic therapy in a rat skin wound infection model. This successful example will provide an effective strategy for the rational design of easy-to-use and time-saving bacterial differentiation agents for potential clinical applications. Moreover, it will stimulate the development of high-performance antimicrobial materials to combat the increasingly serious problem of bacterial infections.

It is worth noting that TTVP is particularly effective in inactivating Gram-positive bacteria, including Staphylococcus aureus, which is one of the main pathogens of wound infections in the human body and others. Thanks to its properties, it may be a promising alternative in the treatment of antibiotic-resistant bacterial infections, which are increasingly becoming a serious clinical problem [63,64].

### 3.2. The Selected PDT Clinical Trials in Head and Neck Tumors

PDT is an effective method of treating early cancers of the oral cavity, pharynx, and larynx, while maintaining normal vital functions. Studies have shown that PDT can be effective in the treatment of head and neck cancer, achieving high rates of cure and clinical response. For example, studies in CIS and T1N0 oral cancer patients after single PDT therapy demonstrated a complete pathological and clinical response, with a 5-year cure rate of 100%.

Similar successes have also been observed in the treatment of more advanced cases of head and neck cancers with PDT. Research results suggest that PDT may be an effective alternative or complement to conventional cancer treatment methods. However, there is a need for further clinical trials, including randomized controlled trials, to more comprehensively evaluate the effectiveness and potential of PDT in the treatment of head and neck cancers [65,66].

### 3.3. The Selected PDT Clinical Trials in the Digestive System Tumors

Photodynamic therapy has proven to be an effective method of treating various types of gastrointestinal cancers, such as Barrett’s esophagus, dysplasia, early cancer, and pancreatic and stomach cancer. In the case of Barrett’s esophagus, PDT is considered an alternative to surgery, especially in cases of high-grade dysplasia. Clinical studies have shown the high effectiveness of PDT in removing dysplasia and preventing progression to invasive cancer [67].

In the case of esophageal cancer, PDT may be effective, especially in the early stages of the disease. However, PDT may be less effective in tumors with a greater depth of penetration. Despite promising results, there are some challenges associated with PDT, such as stricture formation, skin phototoxicity, chest pain, and nausea [65].

In addition to the esophagus, PDT has been used to treat pancreatic cancer, stomach cancer, and colorectal polyps. Despite the experimental nature of these applications, the results of clinical trials are promising. However, further studies are still needed to confirm the effectiveness of PDT in these indications and to optimize its treatment protocols [68,69].

#### 3.3.1. The Selected PDT Clinical Trials in Prostate Cancer

Patients with prostate cancer have limited salvage treatment options after definitive radiotherapy. PDT may be a promising alternative because it provides visible light to selectively destroy cancer cells while minimizing damage to normal tissues. Clinical trials using various photosynthesizers, such as temeporfin and padoporphins, have shown promising results in the treatment of a local recurrence of prostate cancer after radiotherapy. Despite some side effects, such as stress urinary incontinence and urinary rectal fistula, PDT demonstrated the ability to obtain pathological and biochemical responses. Further studies are necessary to evaluate the effectiveness and long-term outcomes of PDT as a salvage therapy for prostate cancer [70,71].

#### 3.3.2. The Selected PDT Clinical Trials in Bladder Cancer

Bladder cancer, especially in its superficial form, can be effectively treated with PDT. Therapeutic response rates ranging from 50% to as high as 80% have been reported in clinical trials using various photosensitizers such as HpD and sodium porfimer. Although disease recurrence is common, PDT has shown promising results in the treatment of both superficial bladder cancer and refractory CIS. Comparative studies with BCG suggest that PDT may be equally effective. Combining PDT with other therapies, such as immunotherapy or chemotherapy, may further increase its effectiveness. Although PDT is approved in some countries, including Canada and some EU countries, its use in the treatment of bladder cancer still requires further research [72,73,74].

#### 3.3.3. The Selected PDT Clinical Trials in Brain Tumors

PDT as an adjunctive therapy for brain tumors has undergone intensive clinical trials. It shows promising results, especially in the case of newly diagnosed and recurrent brain tumors, due to the high uptake of photosensitizers. Clinical trials with HpD, ALA, and temeporfin have shown good therapeutic results, although disease relapses have been observed. PDT used as an adjuvant therapy has shown favorable results in long-term follow-up, but its effectiveness compared to other therapies has not yet been fully established. Research on PDT as a diagnostic and therapeutic tool has shown encouraging results, but more research is needed to determine its ultimate role in the treatment of brain tumors [65,75].

#### 3.3.4. The Selected PDT Clinical Trials in Breast Lumps

Breast cancer is a common health problem among women, often associated with various genetic susceptibilities. However, there are cases where standard treatment strategies do not produce the expected results or lead to side effects. Therefore, new therapeutic approaches rely on innovative solutions such as biomimetic hydrogels. These gels contain chemicals such as tegafur (TF) and protoporphyrin IX (PpIX), which are sensitive to reactive oxygen species (ROS).

Biomimetic hydrogels combine chemotherapy and PDT, which aims to minimize side effects and increase the effectiveness of treatment. During photodynamic therapy, PpIX acts as a photosensitizer which, when activated by light, emits reactive oxygen species, leading to damage to cancer cells. Tegafur, in turn, is a prodrug of fluorouracil, a substance used in chemotherapy. Together, these two substances can interact synergistically to increase the effectiveness of killing cancer cells.

The use of biomimetic hydrogels offers hope for the development of more precise and effective therapies for breast cancer patients while minimizing the side effects associated with traditional treatment methods. These innovative approaches could usher in a new era in cancer therapy, allowing for a more effective and personalized breast cancer treatment [76,77].

#### 3.3.5. The Selected PDT Clinical Trials in Lung Cancer

PDT is increasingly recognized as an important alternative to palliative chemotherapy or radiotherapy for this type of cancer, demonstrating a high response rate of approximately 87% and contributing to improving the quality of life of patients [78].

Studies conducted on lung cancer cells have shown that PDT using a nanobody complex (Nb@IC-NP) effectively destroys cancer cells and improves survival in mice. Additionally, PDT therapy may be used in combination with chemotherapy, radiation therapy, and surgery to treat lung cancer. Potentially more favorable toxicity profiles of photosensitizers such as temoporfin, 2-[1-hexyloxyethyl]-2-devinylpyropheophorbide-a, and chlorine e6 are also indicated [79,80].

Although PDT, especially with Photofrin, may be useful in the palliative treatment of lung cancer, its effectiveness decreases in advanced cancers. Therefore, it is proposed to use new photosensitizers, such as talaporfin and HPPH, which are characterized by a higher absorption at longer wavelengths, which may increase the effectiveness of PDT therapy. These innovative approaches may open new possibilities in the treatment of lung cancer, contributing to improved therapeutic outcomes and quality of life for patients [81].

#### 3.3.6. The Selected PDT Clinical Trials in Skin Cancer

The PDT method is commonly used in the treatment of skin diseases, especially non-melanoma cancers and precancerous skin lesions. According to reports, over 10 million patients have undergone this type of therapy. For actinic keratosis (AK), PDT is usually preferred as the treatment. Studies have shown that PDT based on δ-aminolevulinic acid (ALA) turns out to be more effective than cryotherapy in eliminating lesions [82].

In the treatment of squamous cell carcinoma in situ, the use of PDT with methylaminolevate linate (MAL-PDT) allows for the removal of lesions ranging from 88% to 100%. Although the effectiveness of PDT in basal cell carcinoma is comparable to cryotherapy and surgery, its main advantage is easier application, especially in hard-to-reach areas such as the eye area [83,84].

These are several examples of cancers in which, as has been shown, various types of photosensitizers are used, with varying effectiveness and clinical parameters. Such a wide range of PDT action shows its extraordinary potential in the treatment of many types of cancer—not only those mentioned.

### 3.4. The Selected PDT Research in In Vitro and In Vivo Models

Currently, various in vitro and in vivo tests are being carried out in order to find the best way of treatment. Below, the research results on selected photosensitizers and their action in photodynamic therapy in vitro and in vivo are presented.

In studies conducted on sodium sinoporphyrin, a new photosensitizer isolated from Photofrin, its anticancer potential was assessed both in vitro and in vivo. In clonogenic tests and MTT tests, it was found that sinoporphyrin sodium-PDT is highly effective in inhibiting the growth of tumor cells, compared to Photofrin. The results showed that sinoporphyrin sodium-PDT achieved a clonogenicity inhibition rate of 85.5% to 94.2% at a concentration of 0.5 μg/mL and under irradiation with a wavelength of 630 nm and a power of 30 mW/cm^2^ for 180 s. For MTT tests, the IC50 of sinoporphyrin sodium-PDT was found to be between 0.1 µg/mL and 0.8 µg/mL, which was lower compared to the IC50 of Photofrin-PDT (0.3 µg/mL to 5.5 µg/mL).

In in vivo studies in xenograft models of esophageal cancer and hepatoma in mice, sinoporphyrin sodium-PDT also demonstrated significant antitumor efficacy. Mice were injected with photosensitizers 24 h before irradiation with a laser with a wavelength of 630 nm and a dose of 60 J. The results showed that sinoporphyrin sodium-PDT, especially at a dose of 2 mg/kg, achieved a tumor mass inhibition index of approximately 90%, which was comparable to Photofrin-PDT at a dose of 20 mg/kg.

In summary, sinoporphyrin sodium-PDT showed high anticancer efficacy both in vitro and in vivo, at lower doses compared to Photofrin, suggesting its potential as a promising anticancer drug [85].

Another aspect of in vitro and in vivo research was to investigate the possible synthesis of the field of nanotechnology with photodynamic therapy. Thanks to advances in nanotechnology, scientists now have access to many different nanometric compounds of various shapes, sizes, and structures. These nanoparticles can be synthesized using various methods, which allows their properties to be tailored to specific applications, especially in biomedicine.

In in vitro and in vivo studies, drug nanocarriers in photodynamic therapy (PDT) have shown promising potential. It turned out that nanoparticles can improve the solubility of photosensitizers (PS), protecting them against degradation. Additionally, nanocarriers can change the biodistribution of PS, prolonging their presence in the blood and increasing specificity towards cancer cells while minimizing systemic toxicity.

However, only a small number of nanopreparations have undergone in vivo testing, which poses a major challenge to their clinical application. Also of concern is the fact that nanoparticles may behave differently in in vitro and in vivo models, which may lead to altered properties and the reduced effectiveness of PDT.

To exploit the therapeutic potential of nanoparticles, it is necessary to thoroughly understand their impact on the body, both at the cellular and molecular levels. In particular, it is important to investigate their effects on inflammatory processes, epigenetics, and various -omics to assess their biosafety.

Finally, the assembly of PS with nanoparticles may affect their physicochemical properties and phototoxicity, which may affect their drug delivery ability. For this reason, before starting biological tests, nanoparticle drug preparations should be carefully examined in terms of their biophysical properties.

In conclusion, although the use of nanoparticles as drug carriers in PDT seems promising, there are still many challenges to overcome before they can be introduced into clinical use. Nevertheless, in vitro and in vivo studies on the biological activity of PS nanoparticles allow us to hope that in the future it will be possible to significantly increase the effectiveness of PDT by using these advanced technologies [86].

## 4. Discussion

### 4.1. Potential Side Effects of Photodynamic Therapy

Photodynamic therapy (PDT) is an effective method of treating various diseases but, like many other medical procedures, it may lead to side effects and complications. One of the most common complications is skin burn in the treated area, especially when excessive exposure occurs or in people with photosensitivity. Patients undergoing PDT may also experience pain and discomfort during and after the procedure and may be more susceptible to sunlight for some time after the procedure, which may lead to sunburn.

Additionally, the skin in the treatment area may be red, swollen, scaly, and itchy. There is also a risk of permanent scarring or skin discoloration at the treatment site. The risk of infection in the treatment area is also significant, especially when wounds are not properly cared for and protected.

It is worth noting that the side effects of PDT may vary depending on the type of disease, treatment area, and individual patient response. There is also a risk of more serious complications, so it is important to carry out therapy under the supervision of an experienced specialist who can monitor the patient and respond appropriately to possible complications. The regular assessment of side effects and appropriate care help minimize the risk of complications and improve the effectiveness of PDT therapy [87].

### 4.2. Future Development of Photodynamic Therapy

We are currently seeing a clear and growing interest in photodynamic therapy (PDT) around the world, the result of an intense international research effort. Researchers focus on developing new photosensitizers, investigating PDT mechanisms at the molecular level, increasing effectiveness through combined therapies, and assessing potential clinical indications. Although there are regulatory approvals for the clinical use of PDT photosensitizers and light applicators in many countries, the overall number of approved clinical indications remains limited.

Forecasts suggest that the pharmaceutical industry and research institutes will continue numerous clinical trials to evaluate the applications of PDT, both as a complement to and as an alternative to traditional methods of treating both oncological and non-oncological diseases. Optical methods and nanotechnology play a key role in the characterization of target tissues, determining PDT doses and assessing the results of therapy.

The further investigation of combination therapies, personalized treatment planning, and dosimetry will be necessary and will continue to be important elements of photodynamic therapy. By developing these areas, we can expect further refinement and expansion of PDT applications, which may bring new treatment options and improved therapeutic outcomes for patients.

### 4.3. Assessment of the Effectiveness of PDT Therapy

It is worth emphasizing the significant benefits and positive results of photodynamic therapy (PDT) in the treatment of various diseases. First of all, research confirms the high effectiveness of PDT in the fight against skin cancers, including basal cell carcinoma and squamous cell carcinoma, and in the treatment of precancerous skin lesions such as actinic keratosis. The areas that were analyzed showed significant clinical improvement after the use of PDT, with a visible reduction in the size and number of cancer lesions and a reduction in recurrences.

Additionally, the results suggest that PDT may be an effective therapeutic option for other diseases such as bacterial infections. A positive assessment of the effectiveness of PDT in these areas opens the door to further research on its use in various fields of medicine.

It is also worth emphasizing that PDT often has a favorable safety profile, with minimal side effects and complications compared to other treatment methods, which may improve the quality of life of patients.

Overall, the high potential of PDT as a valid alternative or complement to traditional therapies should be emphasized, encouraging its further use in clinical practice and identifying areas that require further exploration and optimization.

## 5. Conclusions

This work presents the history of photodynamic therapy, starting from ancient times, and the exposure of the body to sunlight, through modern times and ending with contemporary achievements. APDT, which targets diseases caused by bacteria, is also briefly described.

The mechanism of action of photodynamic therapy is described, with particular focus on photosensitizers, as well as their use in various fields of medicine and their potential preparation. Also included are in vivo and in vitro studies focusing on the latest aspects of the use of photodynamic therapy in medicine using the latest photosensitizers and nanotechnology. The work contains a collection of selected cancers and a discussion of the effectiveness of their treatment and the photosensitizers used for this purpose. The problem of the need for further research and development in some of them is also presented.

Despite the significant development of PDT, there is still room for development in the more effective use of photosensitizers or eliminating the potential side effects of this therapy.

## Figures and Tables

**Table 1:** The most popular photosensitizers.

Name	Clinical Application
Hemoporphyrin derivative (HpD) [27]	It is characterized by better selectivity towards tumors than Hp and less photosensitization of the skin.
Sodium porfimer (Photofrin) [28]	It is used in the treatment of many types of cancer, but its main use is in the fight against lung, esophageal, and bladder cancer.
5-Aminolevulinic acid (ALA) [29]	Using this photosensitizer, rare squamous cell carcinoma or prostate cancer is treated.
Methylamine levulinate hydrochloride (MAL) [30]	Its main use is the treatment of basal cell skin cancer.
Verteporfin (Visudyne) [31]	It has a unique application because it is used in the treatment of macular degeneration (AMD) and classic subretinal lesions (CNV).
Hexylamine levulinate hydrochloride (HAL) [32]	Its basic tasks include the diagnosis and subsequent treatment of bladder cancer, but it can be used to treat common skin diseases, such as acne.
Chlorin e6 [33]	It belongs to the more universal types of photosensitizers and has significant activity in the treatment of cervical cancer. Research shows that it may be an effective solution in the treatment of tumors with biotin receptors.
Bacteriochlorophyll [34]	It is a photosensitizer that actively participates in innovative research in the treatment of various types of pathogenic microorganisms, such as Gram-positive bacteria, Gram-negative bacteria, or yeast.
Foscan (Temoporfin) [35]	This photosensitizer is used in the treatment of skin cancer and anatomical structures of the head and neck.
3-Devinyl-3-(1′-hexyloxyethyl) pyrylporphyrin-a (HPPH)- [36]	Used in the treatment of various types of cancer; however, the best results are achieved in the treatment of lung cancer.
